# Problematic Internet Use as a Predictor of Eating Disorders in Students: A Systematic Review and Meta-Analysis Study

**DOI:** 10.3390/nu11092151

**Published:** 2019-09-09

**Authors:** Francisco-Javier Hinojo-Lucena, Inmaculada Aznar-Díaz, María-Pilar Cáceres-Reche, Juan-Manuel Trujillo-Torres, José-María Romero-Rodríguez

**Affiliations:** Department of Didactics and School Organization, University of Granada, 18071 Granada, Spain

**Keywords:** Internet addiction, eating disorders, students, systematic review, meta-analysis

## Abstract

Problematic Internet use (PIU) has begun to be linked to the development of certain eating disorders. This uncontrolled use of the Internet is mainly found in the student population. The purposes of this paper were to determine PIU-related eating disorders in students from a systematic review of the literature and to analyze the incidence of PIU in eating disorders through a meta-analysis of the literature. We used two electronic databases (Web of Science and Scopus) from inception to June 2019. The systematic literature review was based on fixed inclusion and exclusion criteria. A total of 12 studies were identified (systematic review) and 10 studies for meta-analysis, which included 16,520 students. Different eating disorders were associated with PIU: anorexia nervosa, bulimia nervosa, binge-eating disorder, food preoccupation, loss of control eating, and dieting. Furthermore, meta-analysis confirmed that PIU is a predictor of eating disorders in students. The groups of students with PIU presented a higher rate in the presence of eating disorders, these differences being significant. Finally, this study showed empirical evidence on the link between PIU and eating disorders. The need for prevention in childhood and adolescence is highlighted.

## 1. Introduction

Uncontrolled use of technology is a common practice among younger people. The Internet has revolutionized ways of interacting with the world. This fact together with the portability of electronic devices has enabled the proliferation of cases of problematic Internet use (PIU) or Internet addiction.

The PIU has been catalogued within the so-called behavioral addictions [1]. This kind of addiction directly affects the user’s behavior, where there is a loss of self-control and an increase in impulsivity. These factors often cause the user to connect unconsciously to the Internet.

On the other hand, the development of smartphones and social networks have considerably increased the level of internet addiction among university students [2,3,4]. This casuistry mainly affects this population group, increasing the anxiety, depression, and stress levels of students [5,6]. Thus, Internet addiction is a cause of disease in the population [7]. This problem has a global perspective, and mainly affects developed countries.

In childhood it also entails different problems such as a decrease in attention to learning [8], and problematic behaviors such as loss of self-control [9]. It is also a factor related to some eating disorders [10]. In this regard, some years ago several works began to include Internet addiction and eating disorders among problematic behaviors in childhood [11]. Therefore, prevention in the child population is fundamental to preventing the development of this problem in adolescence and adulthood.

Furthermore, PIU has begun to be linked to a lack of physical activity and obesity [12,13], psychiatric disorders [14,15,16], and eating disorders [17,18,19].

On the other hand, some studies suggest that low sympathy, family, school, or behavioral problems and low emotional stability are associated as predictive factors of Internet addiction [20,21]. However, PIU may be a predictor of eating disorders:

- Being repeatedly connected to the Internet encourages sedentarism [12]. In addition, the possibility of ordering food at home (often fast food) avoids the user having to spend time cooking, so you can dedicate it to be connected to the network. This increases the probability of different eating disorders such as loss of control eating, dieting, and binge-eating disorder [22,23].
- And the frequent use of social networks and the possibility of following famous people (influencers, models, actors, and actresses, among others), affects the self-perception of the person [24]. This occurs through social comparison that can affect the emotional mood of the user [25]. This desire to resemble influential people on social networks can affect the presence of eating disorders such as anorexia nervosa, bulimia nervosa, and food preoccupation [26,27].

As for the topic of Internet addiction, previous works of systematic review have been elaborated with meta-analysis that have analyzed different factors linked to the PIU. Liu, Nie, and Wang [28], analyzed 58 papers on Internet addiction in the East Asian region to assess the effects of group counseling programs, cognitive-behavioral therapy, and sports intervention on Internet addiction. Tokunaga [29], reviewed 247 documents to determine correlations between Internet habits and loneliness and depression. Wang et al. [30], analyzed 15 studies to determine the association between Attention Deficit/Hyperactivity Disorder (ADHD) and Internet addiction. Cheng et al. [31], reviewed 25 papers to determine the association between PIU and suicide. Fumero et al. [32], analyzed 28 documents to establish psychological, personal, and social factors related to PIU in adolescents. Lei et al. [33], analyzed 76 documents to determine the relationship between social support and Internet addiction in China. Lei et al. [34], reviewed 77 papers to analyze whether different coping styles correlate with increased Internet addiction. Li et al. [35], included 70 papers in their review on the prevalence of PIU and its associated factors in Chinese university students. Li, Lei, and Tian [36], reviewed 79 studies to explore the relationship between positive and negative indicators of parenting style and Internet addiction. Shao et al. [37], analyzed 26 documents to estimate the prevalence of Internet addiction among university students in China. Su et al. [38], reviewed 101 papers to see if there were significant gender differences in the prevalence of PIU. Lei, Chiu and Li [39], analyzed 70 papers to determine whether the PIU was related to subjective well-being, life satisfaction, positive emotions, or negative emotions.

Although PIU or Internet addiction has begun to link with eating disorders, no previous systematic review study with meta-analysis has addressed these two variables together. In addition, the population at risk of problematic use of the Internet are students [40]. At the same time, eating disorders are more frequent in the student population [41]. All this has repercussions on the incidence of one variable on another. A cause-effect relationship or feedback is generated between these two factors.

## 2. Methods

It was proposed as objectives, (i) to determine PIU-related eating disorders in students from the systematic review of the literature and (ii) to analyze the incidence of PIU in eating disorders through meta-analysis of the literature. Based on them, the research questions were:

RQ1. How many studies were published over the years?
RQ2. Who are the most active authors in the area?
RQ3. In which sources appear this kind of studies?
RQ4. What is the educational stage and the country of the students?
RQ5. What are the eating disorders related to PIU?
RQ6. Is there a significant difference in the presence of eating disorders between the control population and those with PIU?

For this purpose, a systematic review methodology with meta-analysis was used [29,42,43]. In the methodological process, the quality standards of the Preferred Reporting Items for Systematic Reviews and Meta-Analyses (PRISMA) have been applied [44].

The analysis variables were established according to the systematic review methodology [45], and answered the research questions posed. These were: years of publication, authors, source title, characteristics of the students, and eating disorders. On the other hand, the meta-analysis calculated the size of the effect of the set of studies with respect to three assumptions: (i) subjects with PIU have a higher rate of eating disorders; (ii) subjects with non-PIU have a higher rate of eating disorders; (iii) there is no difference between the PIU and non-PIU group in the presence of eating disorders.

### 2.1. Search Strategy

The search was established from the application of different descriptors that defined the topics of the review: (“Problematic Internet use” OR “Internet addiction”) AND (“Eating disorder” OR “Disordered eating” OR “Feeding disorders”). The Web of Science (WOS) and Scopus databases were selected because they have internationally recognized impact indices: Journal Citation Reports (JCR) and Scimago Journal & Country Rank (SJR) [46].

The descriptors were applied in the search engine of both databases to proceed to subsequent filtering. To this end, a series of inclusion and exclusion criteria were established [47].

The inclusion criteria were:

- IC1: Journal articles.
- IC2: Empirical research.
- IC3: The papers are written in English or Spanish language.
- IC4: Association of Problem Internet Use with an Eating Disorder.
- IC5: Population are students.

And the exclusion criteria were:

- EX1: Proceedings of congresses, book chapters, books, or other types of non-peer-reviewed publications.
- EX2: Theoretical studies or reviews.
- EX3: The papers are not described in English or Spanish.
- EX4: The Problematic Internet use is not associated with a particular eating disorder.
- EX5: The study population are not students.

To avoid bias in study selection, two investigators undertook the systematic review using the same descriptors and inclusion and exclusion criteria. The degree of agreement on the inclusion of articles was 98%. The disagreement was addressed by a third researcher who chose to include 100% of the extracted scientific literature.

### 2.2. Data Collection and Analysis

**Figure 1:**
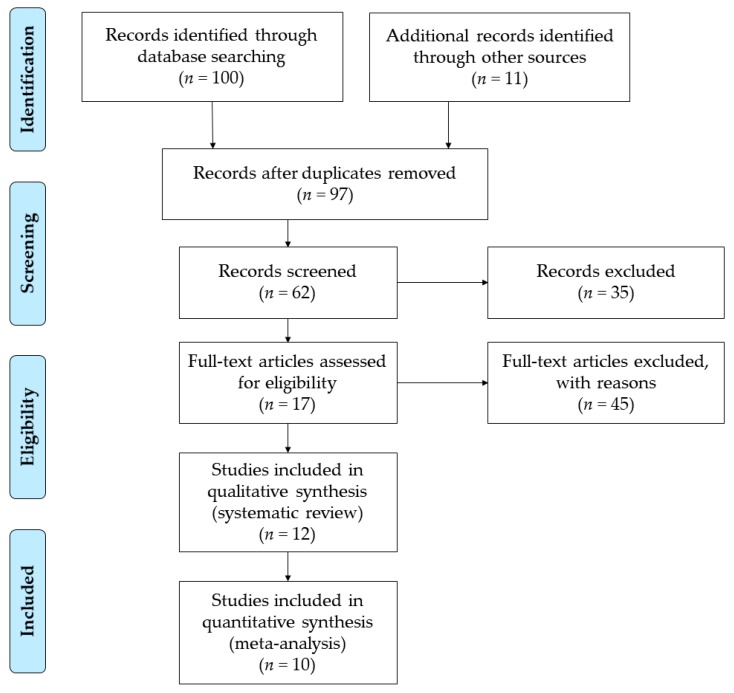
Flowchart according PRISMA Declaration.

The literature screening process was carried out in four phases following the standards of the PRISMA declaration [44] (Figure 1). The first phase “Identification” consisted of the initial search of the literature in the WOS (*n* = 21) and Scopus (*n* = 79) databases, and in the identification of other documents from the references cited in the articles. All references were then extracted and duplicate citations deleted. In the second phase, ‘Screening’, the inclusion (IC1, IC2, IC3) and exclusion (EX1, EX2, EX3) criteria were applied. In the third phase, "Eligibility", each title and summary of the articles was analyzed in detail on the basis of the inclusion (IC4, IC5) and exclusion (EX4, EX5) criteria. Finally, in the fourth phase, "Included", the study sample was included for systematic review (*n* = 12) and meta-analysis (*n* = 10).

Data analysis from the systematic review was performed on the basis of extraction of key information for each analysis variable. This was done by means of a detailed reading of the documents. On the other hand, in the meta-analysis configuration, we included the articles that contained the data referring to the mean and standard deviation of the comparison between a group with PIU and another non-PIU group regarding the presence of eating disorders. Articles that collected dichotomous data regarding the number of eating disorder events for each group (PIU and non-PIU) were also included in the meta-analysis. Only two articles were excluded from meta-analysis because they did not collect this type of data in the text.

Meta-analysis data were analyzed using Review Manager software (Cochrane, London, UK) version 5.3. The confidence interval was set at 95%. The search was conducted on 5 June 2019. All articles published to date were reviewed.

## 3. Results

After filtering the literature, we established the analysis of 12 articles in the systematic review [48,49,50,51,52,53,54,55,56,57,58,59] and 10 in the meta-analysis [48,49,50,52,53,54,56,57,58,59]. The 10 meta-analysis studies were divided according to the type of data provided. Two forest plots were configured, one with studies collecting continuous data (*M, SD*) and the other with studies presenting dichotomous data (number of events detected in the total sample).

### 3.1. Systematic Review

**Figure 2:**
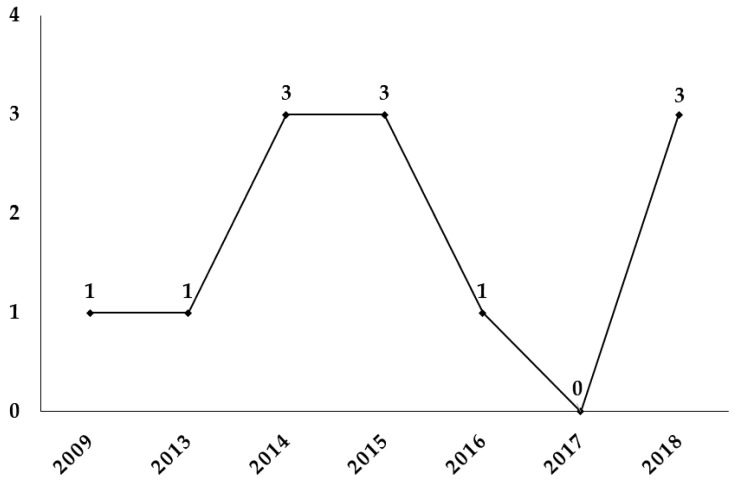
Number of articles per year.

The studies analyzed were grouped according to the year of publication (Figure 2). Of the total number of articles, most were published in 2014, 2015, and 2018 (75%). The start of the topic took place in 2009 and no publications were found between the years 2010–2012 and later in 2017. The years 2009, 2013, and 2016 had a minimum publication fee.

On the other hand, most of the articles are written by multiple authors. Only one of them is of unique authorship [58]. The topic of eating disorders in students has been addressed primarily by different authors who submit only one manuscript (Table 1). Only four authors appear in two separate articles.

Regarding the sources of publication (Table 2), the journal with the most publications on the topic is *Eating and Weight Disorders* (33.33%). The other titles concentrate only one article. The greatest impact, based on h-index, is presented in the *International Journal of Environmental Research and Public Health *(IJERPH) (*h*-index = 68) and the lowest impact in Archives of Medicine (*h*-index = 7). The other journals present an average of 34.88 points in the *h*-index.

In terms of student characteristics (Table 3), most studies analyze eating disorders in college students (83.33%). To a lesser extent there are three studies that focus on the High School stage (25%) and one study that analyzes the Middle School stage (8.33%). The average age of students in the university stage ranges from 18.55 to 27.61 years old (*M *= 21.32; *SD* = 2.38). The countries from which the students come are Turkey (25%), Spain (16.66%), China (16.66%), the USA (16.66%), Colombia (8.33%), Egypt (8.33%), and Taiwan (8.33%). The total sample size was 16,520 subjects. Among the different studies the sample ranged from 314 to 2365 students (*M* = 1377; *SD* = 784).

In relation to the eating disorders associated with each study (Table 4), bulimia nervosa is the disorder with the most cases (91.66%). Furthermore, highlights the interest in anorexia nervosa (50%), food preoccupation (41.66%), loss of control eating (41.66%), dieting (41.66%), and binge eating disorder (16.66%). To evaluate these eating disorders, studies have mainly used the Eating Attitude Test-26 (EAT-26) (41.66%), the SCOFF scale (Sick, Control, One, Fat, Food) (25%), the eating disorder inventory (EDI-1) (16.66%), Borderline Symptom List (BSL-23) (8.33%) and the Eating Disorder Examination Questionnaire (EDEQ) (8.33%). The reliability achieved in each of the studies was good: 0.86 in EAT-26 [48]; 0.94 in EAT-26 [49]; 0.85 in SCOFF [50]; 0.70 in EAT-26 [51]; 0.98 in SCOFF [52]; 0.64 to 0.82 in BSL-23 [53]; 0.88 in EAT-26 [54]; 0.90 in EAT-26 [55]; 0.80 in SCOFF [56]; 0.95 in EDEQ [57]; 0.88 in EDI-1 [58]; 0.84 in EDI-1 and 0.81 in EAT-26 [59].

### 3.2. Meta-Analysis

**Figure 3:**
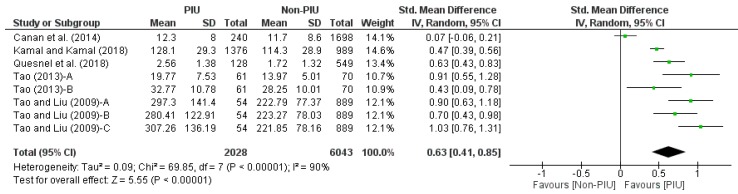
Forest plot of meta-analysis with continuous data.

Two meta-analyses were made based on the data presented by the scientific articles. Meta-analysis with continuous data consisted of five studies [49,54,57,58,59] (Figure 3). In Tao [58] and Tao and Liu [59] all the comparisons between groups with PIU and groups with non-PIU (differentiated in the forest diagram with the letters A, B and C) were collected.

Specifically, two assumptions were made regarding the effect of groups (PIU and non-PIU) on eating disorders: a majority in favor of the PIU group and a single study that had no effect [49]. Thus, most of the investigations were placed to the right of the no-effect line and one of them was on the no-effect line. The diamond figure at the far right confirmed that the difference between groups is statistically significant in favor of the PIU group. This was corroborated in the value that was obtained in the size of the effect (*p* < 0.00001). In addition, according to Cohen’s *d* coefficient, the prevalence of eating disorders in the PIU group had a mean effect size (*d* = 0.63, 95% CI = 0.41, 0.85). As a result, Internet addiction is associated with the presence of eating disorders.

**Figure 4:**
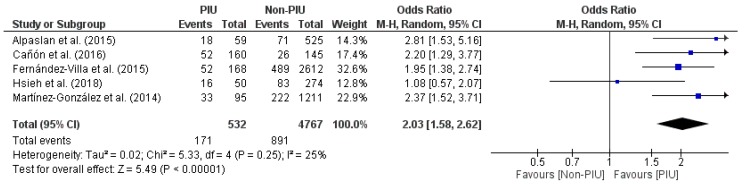
Forest plot of meta-analysis with dichotomous data.

On the other hand, meta-analysis with dichotomous data was composed by five other studies [48,50,52,53,56] (Figure 4). The same two assumptions were repeated as in the previous forest plot: studies with effect in favor of the PIU group and a single study with no effect [53]. The figure of the diamond was placed at the far right confirming the significant difference between groups. The effect size obtained a statistically significant value (*p* < 0.00001). So the second meta-analysis continued to confirm that PIU is related to the presence of eating disorders (OR = 2.03, 95% CI = 1.58, 2.62).

## 4. Discussion

The total number of documents analyzed collected a total sample of 16,520 students. In this large sample of students from different countries and continents, the PIU was linked to the presence of eating disorders. Although PIU is associated with different eating disorders, the published literature on these two variables is limited. However, in 2018 scientific production has begun to resume.

The diversity of authors denotes that there is no specialization in the topic. This confirms that empirical research on PIU and eating disorders is incipient. At the same time, the journals that have published on these two variables are different. The only journal with more than one document is *Eating and Weight Disorders*, which has a total of four.

On the other hand, the educational stage where this phenomenon has been studied the most is university students, coinciding with the fact that they are the population that is most highly at risk [5,6,40]. Thus, attention was focused on that sector of the population, which has the highest rate of PIU. Therefore, the need arises to establish preventive measures in childhood and adolescence to avoid this type of behavior in later stages. From the educational and family spheres, specific actions must be taken to educate students in the proper use of technology.

The variability of countries where research data have been collected confirms the global scale of the problem. So the PIU is not an isolated event from a given region, but is occurring in different countries and continents at an incessant pace [2,3,4].

With respect to the type of eating disorders linked to the PIU, the presence of anorexia nervosa, bulimia nervosa, binge-eating disorder, food preoccupation, loss of control eating, and dieting was detected [17,18,19,26,27]. PIU encourages sedentarism because of the user’s need to be connected to the Internet as often as possible [12,13]. This leads to neglect of healthy food and high ingestion of junk food, which is easily accessible and quickly processed.

On the other hand, another danger associated with PIU is the constant consultation of social networks. This phenomenon has had a direct impact on the way of life and behavior of young people. Social networks such as Instagram broadcast daily thousands of audiovisual contents that have an impact on different users. One of the dangers is the recurrence to social comparison with this type of users who have a high audience and present a stylized body figure [24,25]. This can affect the development of eating disorders such as anorexia nervosa or bulimia nervosa.

Finally, meta-analyses confirmed that students with PIU are more likely to develop eating disorders. Thus the assumption (i) that subjects with PIU have a higher rate of eating disorders is verified. So the PIU is a predictor of eating disorders in the student population.

To sum up, in line with the different previous studies [28,29,30,31,32,33,34,35,36,37,38,39], 12 documents were analyzed that determined the influence of the PIU in the development of different eating disorders.

## 5. Conclusions

The confirmation of the link between PIU as a predictor of eating disorders leads to a new line of research in studies on the development of eating disorders in childhood, adolescence and young adulthood. The population most at risk are university students. Preventive measures should therefore be introduced at lower educational stages.

This paper responded to the objectives of the study insofar as it determined PIU-related eating disorders in students and analyzed the incidence of PIU in eating disorders. In addition, the paper highlights the need for prevention in childhood and adolescence. Therefore, the main practical application is the elaboration of informative material on the risks of suffering a PIU and the implementation of formative sessions in the educational levels of Middle School and High School.

The limitations of the study are highlighted as (i) the limitation of searching scientific documents only in the WOS and Scopus databases, although for a first approximation to the topic of study it is more than sufficient, since the literature with the greatest scientific impact has been analyzed, and (ii) the bias of researchers in selecting articles, which we attempted to resolve with the opinion of a third researcher.

Finally, the data obtained enable the creation of new studies and the continuity of this paper. Confirmation that PIU is a predictor of eating disorders should continue to be investigated in new empirical studies linking these two variables. On the other hand, the analysis of the type of behavior carried out by users on the Internet and how this behavior is linked to specific eating disorders is an interest topic. So that studies emerge that analyze the relationship between the abusive use of social networks with anorexia nervosa and bulimia nervosa. Or the abusive use of video games with dieting and binge-eating disorder.

To sum up, Internet addiction is one of the main challenges facing today’s society. The excessive use of technology is causing different problems in human health. One of them is an increase in eating disorders. Researchers and professionals can help to detect these consequences and propose measures to avoid this type of problematic behaviour.

## Figures and Tables

**Table 1:** Authors’ names and number of publications.

Author	Total
A.J. Molina, T. Fernández, V. Martín, Z.L. Tao	2
A. Almaraz, A. Ataoglu, A. Bueno, A. Hisarvant, A.G. Mabe, A.H. Alpaslan, B. Cook, C. Ayán, C. Gunes, Ç.B. Çelik, C.F. Yen, D.A. Quesnel, D.C. Hoyos, D.R. Leal, E. Jiménez-Mejías, E.A. Sánchez, F. Canan, G. Sinani, H. Odaci, H. Uzel, J. Alguacil, J. Llorca, J. Zamudio, J.C. Jaramillo, J.J. Castaño, J.M. Cancela, K. Avci, K. Murray, K.J. Forney, K.Y. Hsieh, L. Martínez-González, L.F. Vallero-Juan, L.S. Urueña, M. Delgado-Rodríguez, M. García-Martín, N. Bayraktar, N.N. Kamal, N.N. Kamal, O. Yildrim, P.K. Keel, R. Campelo, R. Mateos, R. Ortíz, R. Rincón, R.C. Hsiao, S.C. Cañón, T. Yildrim, T.L. Liu, U. Koçak, Y. Liu, Y.H. Yang	1

**Table 2:** Source title and *h*-index.

References	Journal	*h*-Index
[52]	Adicciones	20
[50]	Archives of Medicine	7
[49]	Cyberpsychology, Behavior, and Social Networking	54
[48,51,58,59]	Eating and Weight Disorders	23
[55]	International Journal of Eating Disorders	49
[53]	International Journal of Environmental Research and Public Health	68
[57]	International Journal of Mental Health and Addiction	23
[54]	International Journal of Preventive Medicine	35
[56]	Nutrición Hospitalaria	35

**Table 3:** Characteristics of the students.

Reference	Educational Stage			*n*	Age (*M*)	Country
	Middle School	High School	University			
Alpaslan et al. (2015) [48]	X	X		584	16.12	Turkey
Canan et al. (2014) [49]		X		1938	16.05	Turkey
Cañón et al. (2016) [50]			X	640	22.15	Colombia
Çelik et al. (2015) [51]			X	314	20.65	Turkey
Fernández et al. (2015) [52]			X	2780	20.5	Spain
Hsieh et al. (2018) [53]			X	500	22.1	Taiwan
Kamal and Kamal (2018) [54]			X	2365	21.0	Egypt
Mabe et al. (2014) [55]			X	1960	18.7	USA
Martínez et al. (2014) [56]			X	1306	19.85	Spain
Quesnel et al. (2018) [57]			X	898	27.61	USA
Tao (2013) [58]			X	2036	20.7	China
Tao and Liu (2009) [59]		X	X	1199	18.9	China

**Table 4:** Eating disorders associated with each reference.

Reference	Anorexia Nervosa	Bulimia Nervosa	Binge-Eating Disorder	Food Preoccupation	Loss of Control Eating	Dieting
Alpaslan et al. (2015) [48]		X		X	X	X
Canan et al. (2014) [49]		X		X	X	X
Cañón et al. (2016) [50]	X	X				
Çelik et al. (2015) [51]	X					
Fernández et al. (2015) [52]	X	X				
Hsieh et al. (2018) [53]		X	X			
Kamal and Kamal (2018) [54]		X		X	X	X
Mabe et al. (2014) [55]		X		X	X	X
Martínez et al. (2014) [56]	X	X				
Quesnel et al. (2018) [57]	X	X	X			
Tao (2013) [58]	X	X				
Tao and Liu (2009) [59]		X		X	X	X
